# Supplementary figures and images for: Giant clams as open-source, scalable reef environmental biomonitors
Source: PLoS One. 2023 Jan 5;18(1):e0278752. doi: 10.1371/journal.pone.0278752 (PMC9815582; doi:10.1371/journal.pone.0278752)

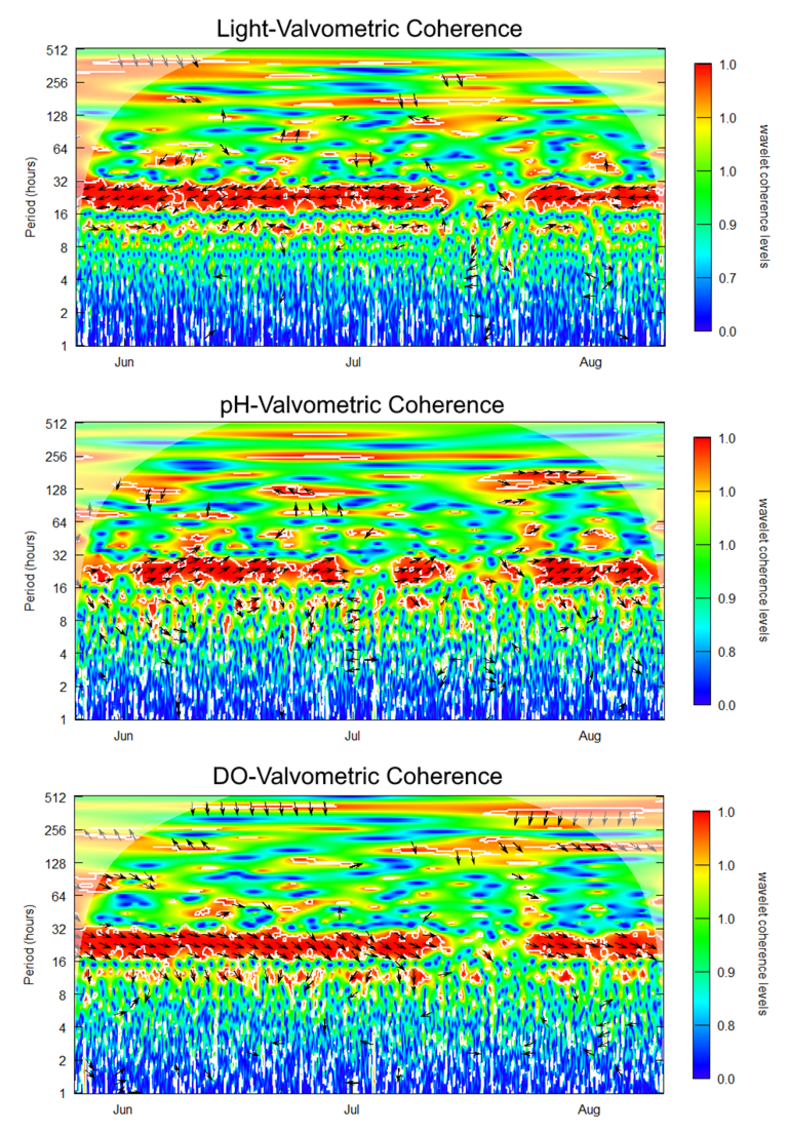

Supplement: S1 Fig — In all three diagrams, we see high coherence levels at the 24-hour and 12-hour periodicities, and less so at the 6-hour periodicity. The arrows refer to areas of significant coherence, with right-pointing arrows referring to relationships in phase, while left refers to anti-phased relationships. Light is anti-phased with valvometric activity as the clams record higher closure values at night when light levels are near zero. pH and DO are in phase. (TIF) [file pone.0278752.s001.tif]

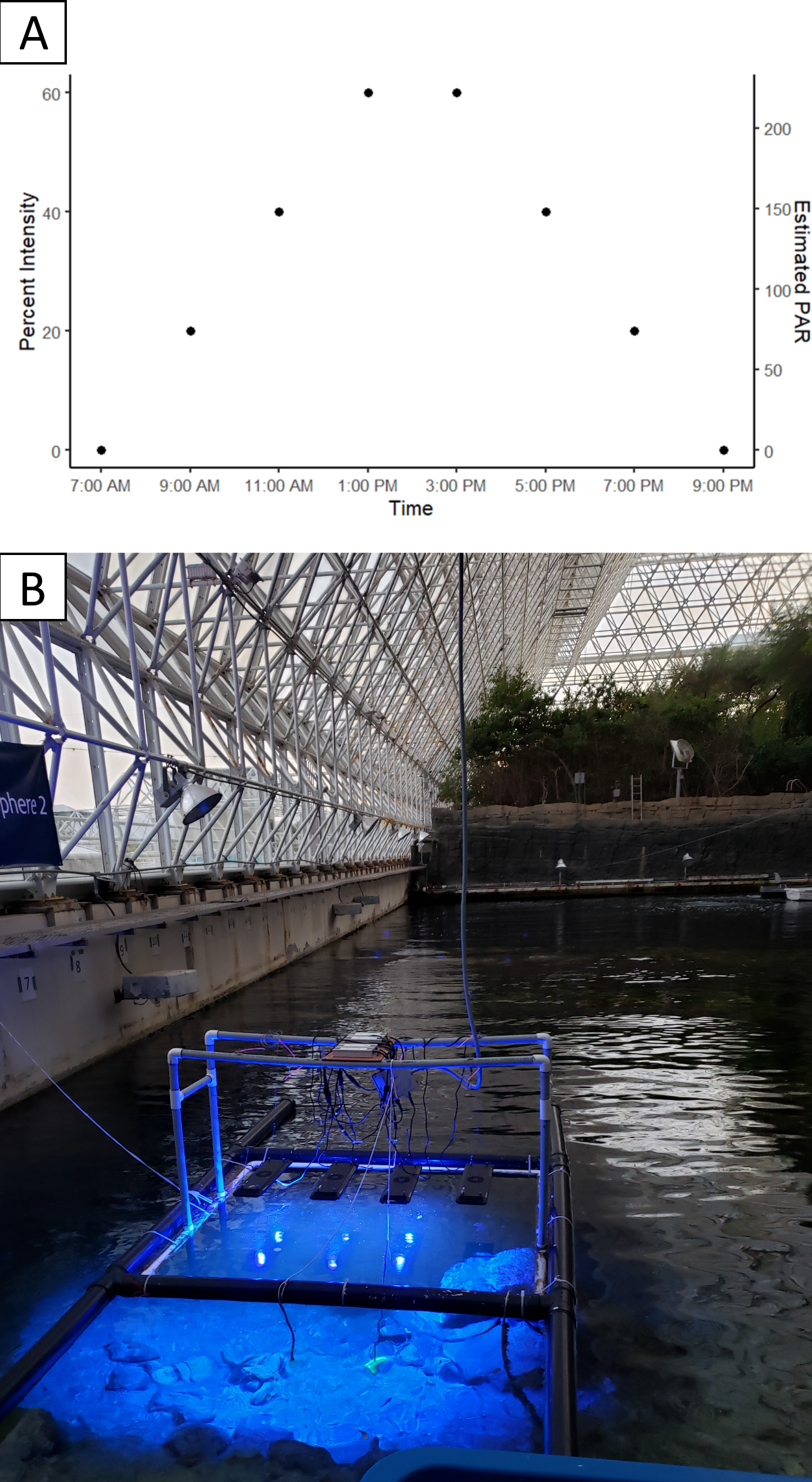

Supplement: S2 Fig — A: Kessil “Tuna Blue” light program, showing the diurnal schedule as % intensity, and approximate PAR levels assuming a peak value of 370 μmoles photons/m2s. B: A view of the floating lighting rig on a cloudy, snowy day. (TIF) [file pone.0278752.s002.tif]

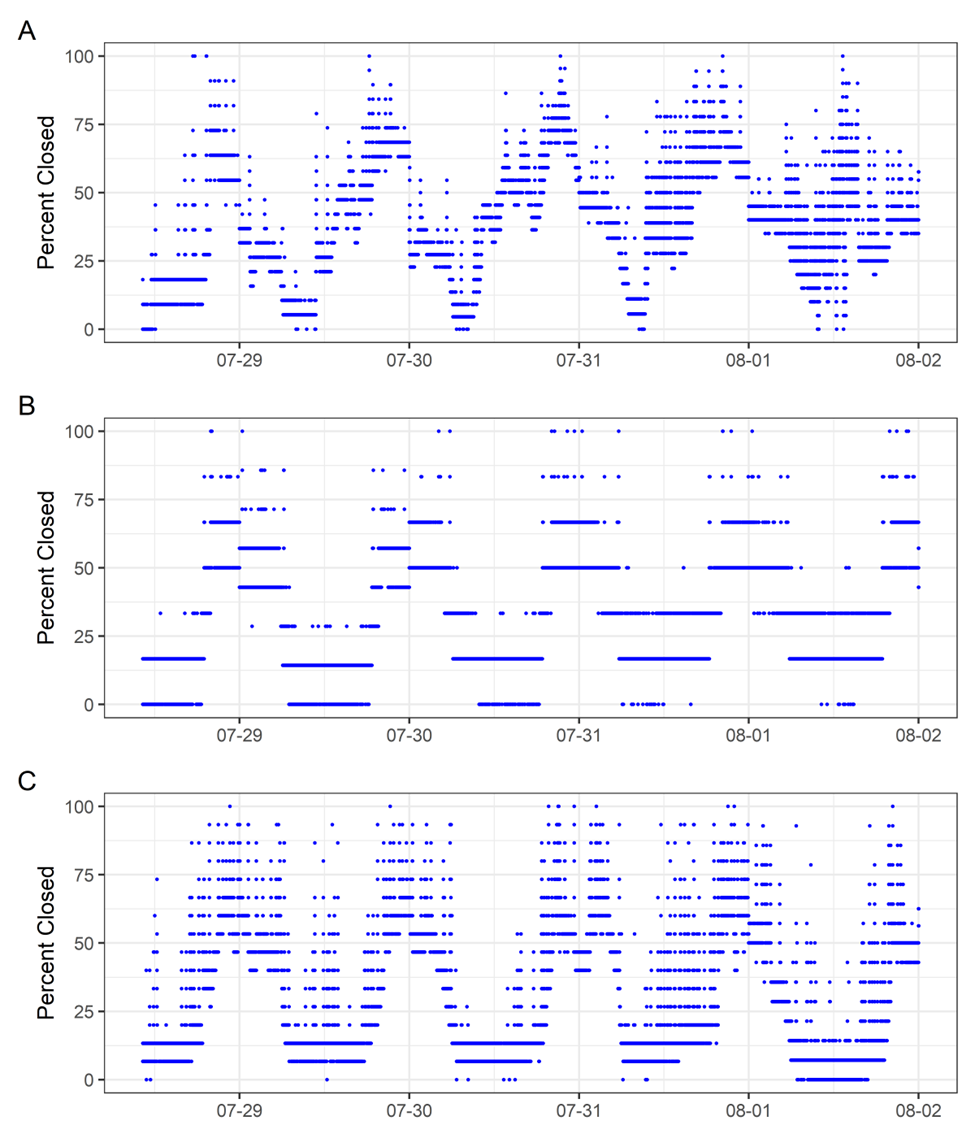

Supplement: S3 Fig — (TIF) [file pone.0278752.s003.tif]

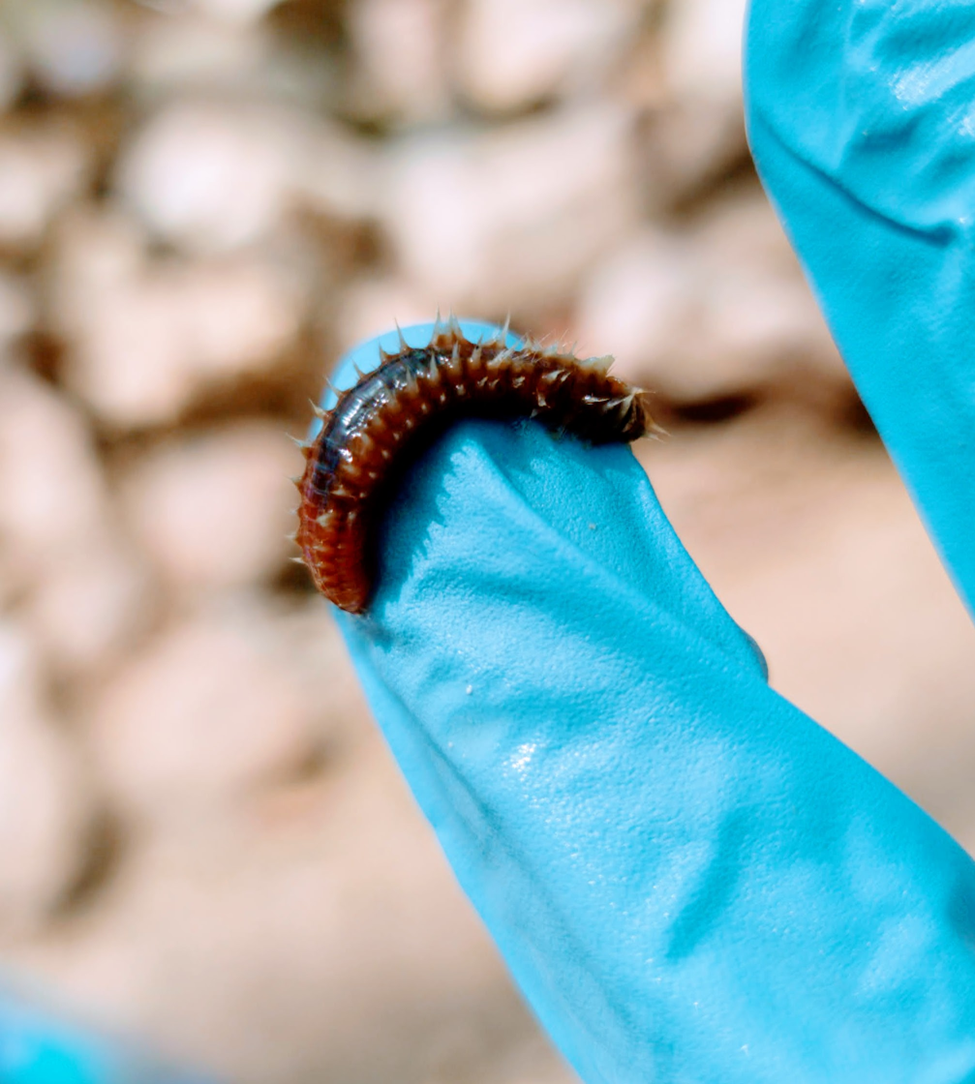

Supplement: S4 Fig — The smaller individuals frequently attempt to attack the clams through the byssal opening. (TIF) [file pone.0278752.s004.tif]
