# Supplementary material for: Giant clams as open-source, scalable reef environmental biomonitors
Source: PLoS One. 2023 Jan 5;18(1):e0278752. doi: 10.1371/journal.pone.0278752 (PMC9815582; doi:10.1371/journal.pone.0278752)

**Supplemental Materials**

**Sensor Construction SOP**

1. Align correct contacts of Hall effect sensor with stripped terminal ends of each braided cable.


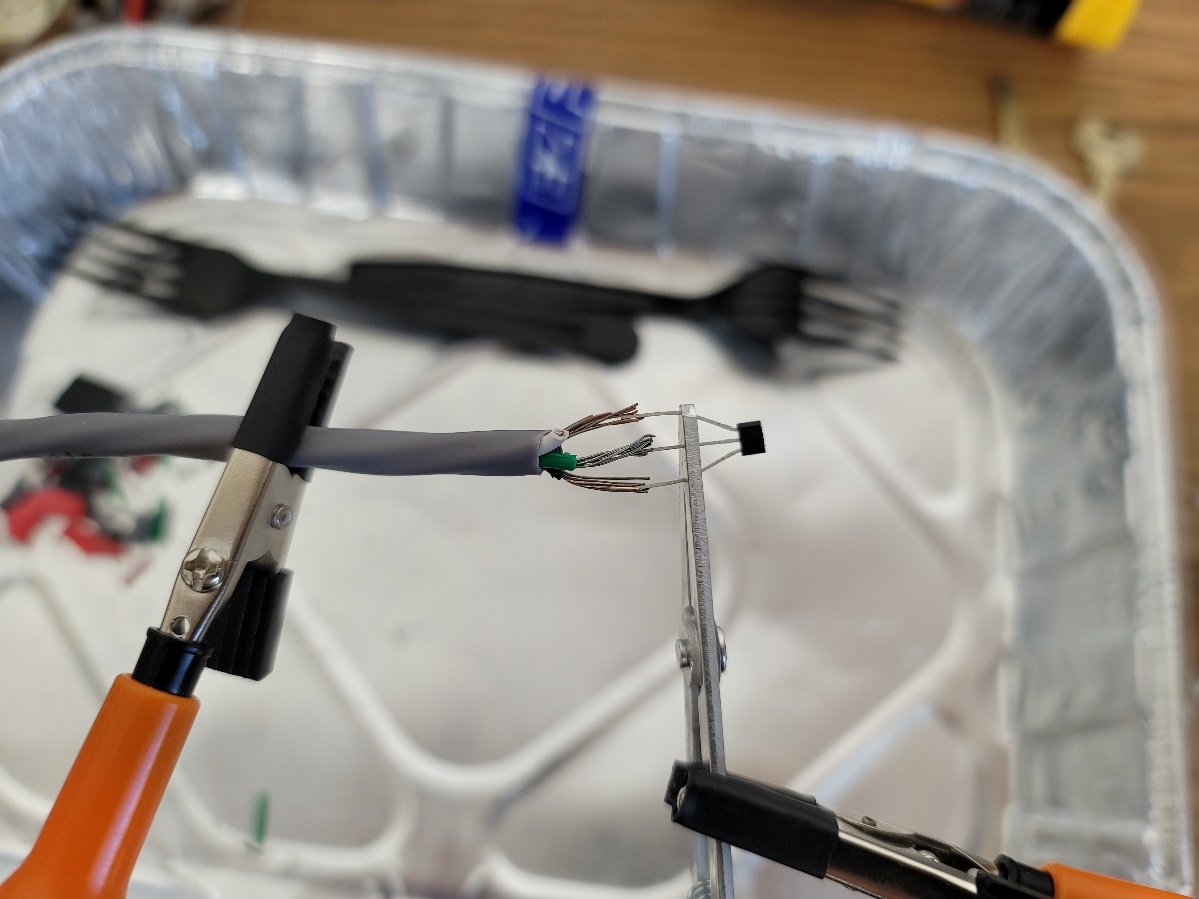


1. Apply solder to bond the contacts to the cables. Note the heat-protection clip used to carry heat from solder away from the sensor.


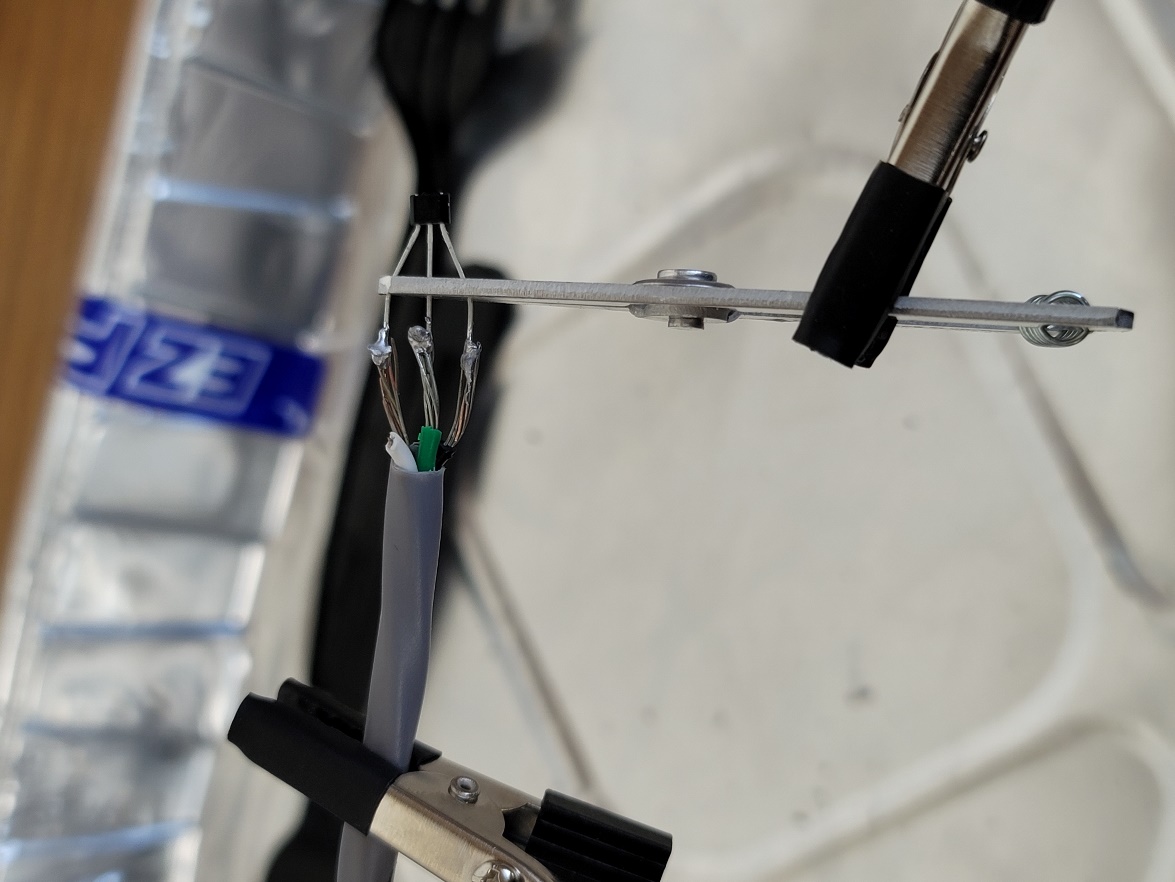


1. Wrap outer welds with heat-stable electrical tape. This will prevent the welds from touching each other and causing a short when the sensor is applied to the clam.


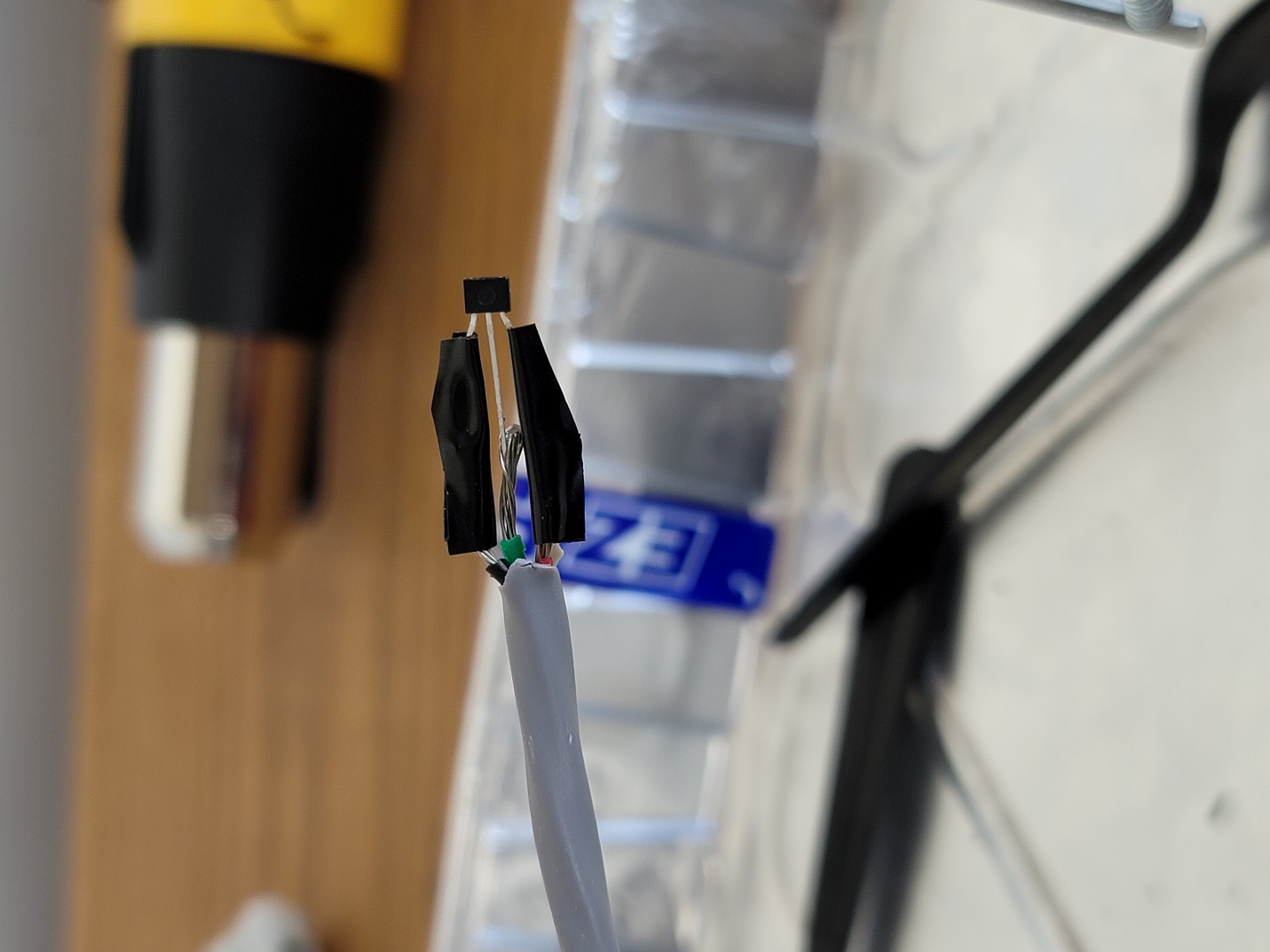


1. Coat the sensor and cable terminal with marine-grade dielectric grease. This provides additional protection against any shorts between contacts and serves as a last line of defense against water intrusion.


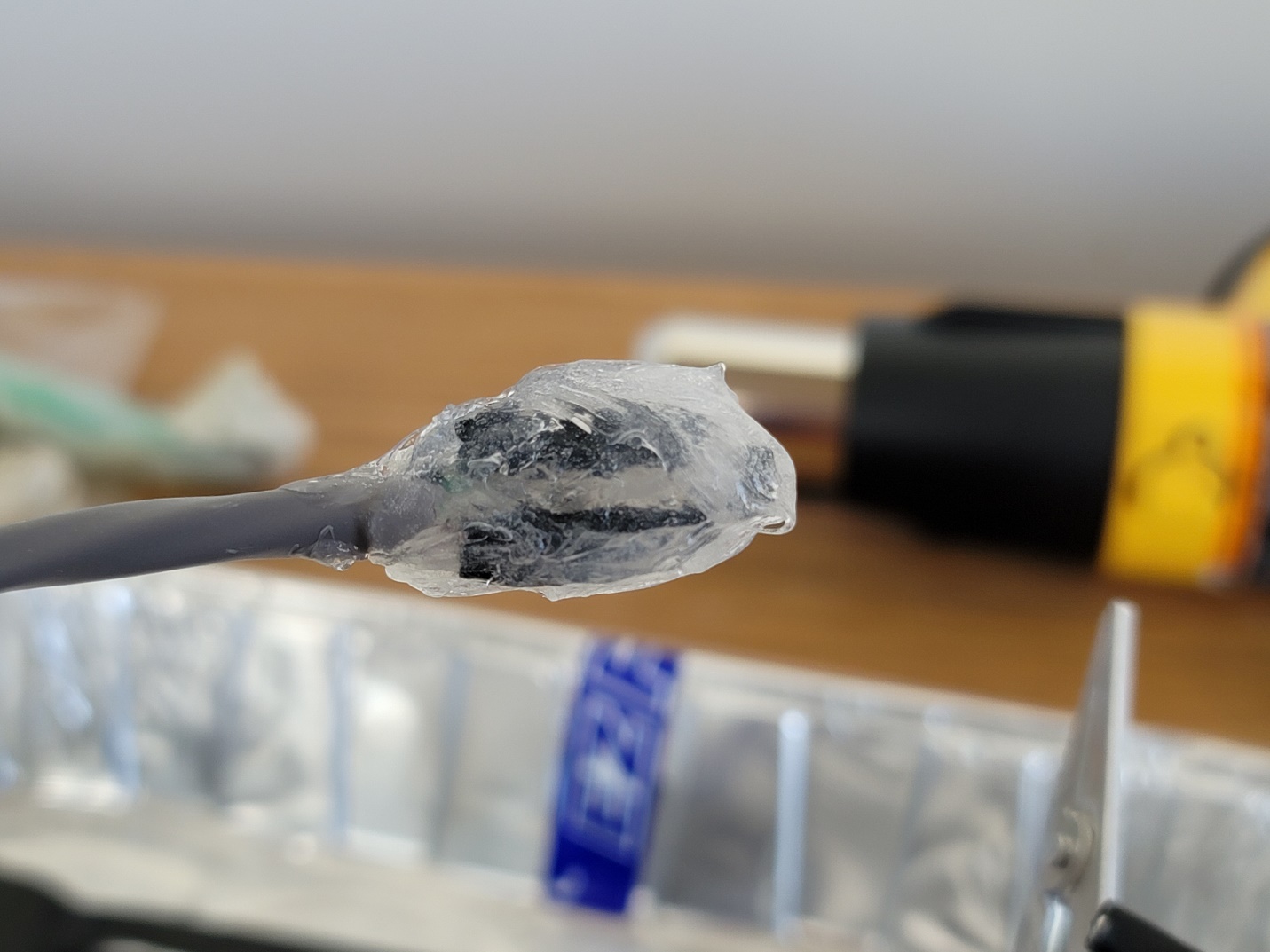


1. Wrap in heat-shrink tubing and constrict with a heat gun.


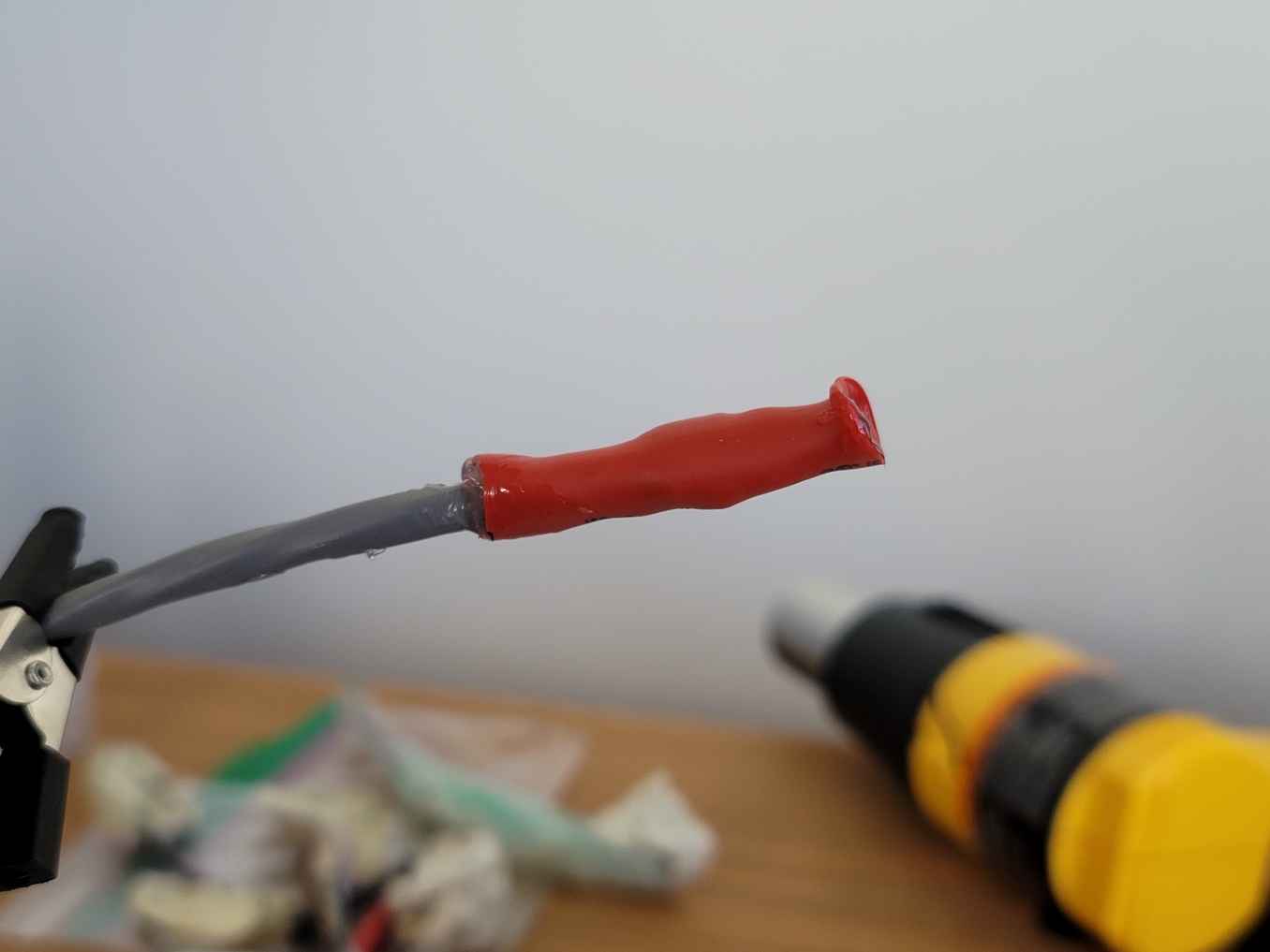


1. Apply aquarium-grade silicone in an even layer, extending to least 1 cm of the original cable. Spread the coat even with a spreading tool. Leave over 24 hours to cure; do not connect to the Arduino until silicone has fully cured. If too thick, the silicone will take an extended time to finish curing inside.


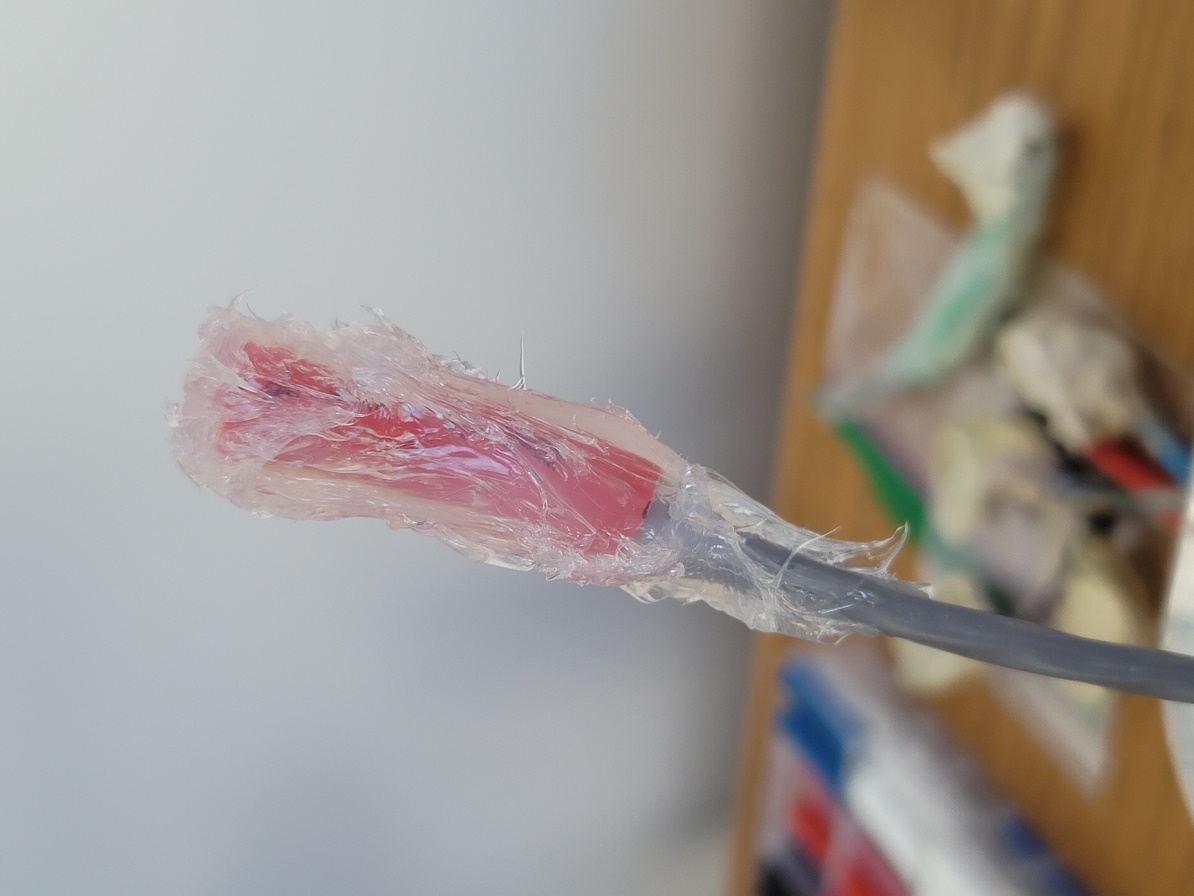

Supplement: S1 File — (DOCX) [file pone.0278752.s005.docx]
