# Supplementary material for: Giant clams as open-source, scalable reef environmental biomonitors
Source: PLoS One. 2023 Jan 5;18(1):e0278752. doi: 10.1371/journal.pone.0278752 (PMC9815582; doi:10.1371/journal.pone.0278752)
Supplement: S1 Data — (ZIP) [file pone.0278752.s006.zip › readme.docx]

“raw valvometric data” is a large CSV with over 2.9 million rows used to inform all following analyses in the study. It includes the following data at 5-second resolution:

- datetime
- Sensor1 through Sensor3: raw mV values measured from each sensor by the Arduino
- zscore1 through zscore3: daily z-scores for sensor 1 through 3

“daily data merged” includes daily-scale data used to conduct GAM analyses. The CSV file includes the following averaged at daily resolution:

- date
- count: number of times per day clam 3 closed its shell
- chlor: daily average chlorophyll-a RFU
- bga: daily average phycoerythrin RFU
- pH
- DO: daily average dissolved oxygen (mg/L)
- light: daily average PAR in levels in μmol electrons/m^2^s

“minute scale valvometry” is a CSV with minute-scale data used to run wavelet analysis (both raw and day-detrended versions included in Figure 7.

- date
- sensor3: valvometric z-scores for clam sensor 3

“Combined 15 minute data” is data used to run wavelet coherence analyses. The data takes the form of a CSV incorporating the following data at 15-minute resolution:

- sensor3: valvometric z-scores for clam sensor 3
- DO: dissolved oxygen (mg/L)
- pH
- chla: chlorophyll-a (Relative Fluorescence Units)
- PE: phycoerythrin (Relative Fluorescence Units)
- light: PAR levels in μmol electrons/m^2^s
