# Supplementary material for: Giant clams as open-source, scalable reef environmental biomonitors
Source: PLoS One. 2023 Jan 5;18(1):e0278752. doi: 10.1371/journal.pone.0278752 (PMC9815582; doi:10.1371/journal.pone.0278752)
Supplement: Attachment — Submitted filename: plos response to reviewers 42922.docx [file pone.0278752.s007.docx]

PONE-D-21-39374

Giant clams as open-source, scalable reef environmental biomonitors

PLOS ONE

Dear Dr. Killam,

Thank you for submitting your manuscript to PLOS ONE. After careful consideration, we feel that it has merit but does not fully meet PLOS ONE’s publication criteria as it currently stands. Therefore, we invite you to submit a revised version of the manuscript that addresses the points raised during the review process.

We have two reviews from two experts in this field. They both suggest major revisions and offer constructive comments - both state the value in this work and I agree with their conclusions.

Please submit your revised manuscript by Apr 23 2022 11:59PM. If you will need more time than this to complete your revisions, please reply to this message or contact the journal office at [plosone@plos.org](mailto:plosone@plos.org). When you're ready to submit your revision, log on to https://www.editorialmanager.com/pone/ and select the 'Submissions Needing Revision' folder to locate your manuscript file.

Please include the following items when submitting your revised manuscript:

- A rebuttal letter that responds to each point raised by the academic editor and reviewer(s). You should upload this letter as a separate file labeled 'Response to Reviewers'.
- A marked-up copy of your manuscript that highlights changes made to the original version. You should upload this as a separate file labeled 'Revised Manuscript with Track Changes'.
- An unmarked version of your revised paper without tracked changes. You should upload this as a separate file labeled 'Manuscript'.

If you would like to make changes to your financial disclosure, please include your updated statement in your cover letter. Guidelines for resubmitting your figure files are available below the reviewer comments at the end of this letter.

If applicable, we recommend that you deposit your laboratory protocols in protocols.io to enhance the reproducibility of your results. Protocols.io assigns your protocol its own identifier (DOI) so that it can be cited independently in the future. For instructions see: <https://journals.plos.org/plosone/s/submission-guidelines#loc-laboratory-protocols>. Additionally, PLOS ONE offers an option for publishing peer-reviewed Lab Protocol articles, which describe protocols hosted on protocols.io. Read more information on sharing protocols at <https://plos.org/protocols?utm_medium=editorial-email&utm_source=authorletters&utm_campaign=protocols>.

We look forward to receiving your revised manuscript.

Kind regards,

David P. Gillikin, Ph.D.
Academic Editor
PLOS ONE

Journal Requirements:

When submitting your revision, we need you to address these additional requirements.

1. Please ensure that your manuscript meets PLOS ONE's style requirements, including those for file naming. The PLOS ONE style templates can be found at

<https://journals.plos.org/plosone/s/file?id=wjVg/PLOSOne_formatting_sample_main_body.pdf>  and

<https://journals.plos.org/plosone/s/file?id=ba62/PLOSOne_formatting_sample_title_authors_affiliations.pdf>

**Thank you. We have submitted this new draft following PLOS ONE’s style.**

2. Thank you for stating the following in the Acknowledgments Section of your manuscript:

“Funding was provided by the Brown Foundation and the University of Arizona Postdoctoral Fellowship (to DK), and University of Arizona Research, Innovation, and Impact (to DMT)..”

We note that you have provided additional information within the Acknowledgements Section that is not currently declared in your Funding Statement. Please note that funding information should not appear in the Acknowledgments section or other areas of your manuscript. We will only publish funding information present in the Funding Statement section of the online submission form.

Please remove any funding-related text from the manuscript and let us know how you would like to update your Funding Statement. Currently, your Funding Statement reads as follows:

 “DK- Brown Foundation, University of Arizona Postdoctoral Fellowship

DT- University of Arizona RII

**We have revised our acknowledgement to not include funding information. The University of Arizona RII grant for Diane Thompson is synonymous with the Research, Innovation and Impact grant, so we have made that clearer in the funding statement.**

The funders had no role in study design, data collection and analysis, decision to publish, or preparation of the manuscript.”

Please include your amended statements within your cover letter; we will change the online submission form on your behalf.

3. We note that you have stated that you will provide repository information for your data at acceptance. Should your manuscript be accepted for publication, we will hold it until you provide the relevant accession numbers or DOIs necessary to access your data. If you wish to make changes to your Data Availability statement, please describe these changes in your cover letter and we will update your Data Availability statement to reflect the information you provide.

**Noted. Upon acceptance we will provide links + DOIs to the relevant Git repository (where Arduino and R processing code will be stored), as well as the University of Arizona and Dryad open data repositories.**

4. Please include captions for your Supporting Information files at the end of your manuscript, and update any in-text citations to match accordingly. Please see our Supporting Information guidelines for more information: <http://journals.plos.org/plosone/s/supporting-information>.

**We have added captions for our SI files to the end of the manuscript.**

[Note: HTML markup is below. Please do not edit.]

Reviewers' comments:

The reviewers provided extensive and helpful feedback and suggestions which we feel have greatly improved the manuscript, both in its understandability and rigor. We have added new supplementary figures, new statistical tests and analysis to the Results section, and have redone several figures to use the “Percent closed” metric recommended by Reviewer 1. We also greatly expanded our methods section to describe in greater detail the rationales we had behind our choices on sensor attachment, as well as the valvometric data processing.

Reviewer's Responses to Questions

Comments to the Author

1. Is the manuscript technically sound, and do the data support the conclusions?

The manuscript must describe a technically sound piece of scientific research with data that supports the conclusions. Experiments must have been conducted rigorously, with appropriate controls, replication, and sample sizes. The conclusions must be drawn appropriately based on the data presented.

Reviewer #1: Partly

Reviewer #2: Partly

2. Has the statistical analysis been performed appropriately and rigorously?

Reviewer #1: No

Reviewer #2: Yes

3. Have the authors made all data underlying the findings in their manuscript fully available?

The [PLOS Data policy](http://www.plosone.org/static/policies.action#sharing) requires authors to make all data underlying the findings described in their manuscript fully available without restriction, with rare exception (please refer to the Data Availability Statement in the manuscript PDF file). The data should be provided as part of the manuscript or its supporting information, or deposited to a public repository. For example, in addition to summary statistics, the data points behind means, medians and variance measures should be available. If there are restrictions on publicly sharing data—e.g. participant privacy or use of data from a third party—those must be specified.

Reviewer #1: Yes

Reviewer #2: Yes

4. Is the manuscript presented in an intelligible fashion and written in standard English?

PLOS ONE does not copyedit accepted manuscripts, so the language in submitted articles must be clear, correct, and unambiguous. Any typographical or grammatical errors should be corrected at revision, so please note any specific errors here.

Reviewer #1: Yes

Reviewer #2: Yes

5. Review Comments to the Author

Please use the space provided to explain your answers to the questions above. You may also include additional comments for the author, including concerns about dual publication, research ethics, or publication ethics. (Please upload your review as an attachment if it exceeds 20,000 characters)

Reviewer #1: In this manuscript, the authors describe a low-cost approach to valveometry for research in fields such as aquaculture and conservation. I am currently conducting valveometry research in my own lab and completely agree with the authors that this is a valuable research approach that is currently plagued by high cost issues. The system they describe seems to be very functional and instructions on how to build it are fairly clear. This part is great. The data they generated seems consistent with the types of data output from higher cost, commercial units (for example, AquaDect MosselMonitors). The authors also do a very nice job of describing some mechanical shortcomings of their system and how to overcome them. These aspects of the manuscript are very useful and will be of strong interest to many readers of this journal.

**We are grateful to have a “valvometrist” among our reviewers, and are pleased that they agree on the demand for inexpensive, open-source valvometric equipment! In many ways, this was the most challenging part of the paper in terms of design and execution. We wanted to ensure that people would not have to reinvent the wheel in replicating our approach.**

The other challenge of valveometry revolves around the tremendous amount of data generated, what endpoints to calculate/analyze from this data, and how to define these endpoints. Even something seemingly as simple as “closure” can be defined, calculated, and analyzed in many different ways and it is not always easy to figure out the best way to do this. This aspect of the manuscript is where things seem to fall short and I have many concerns. The system built by the authors has clearly generated a high volume of potentially useful data, but the analytical approaches and logic often seem to be unclear, weak or absent for the conclusions that the authors have drawn. I give several examples below and hope the authors find them useful. These are not easy datasets to analyze and I am sympathetic to the challenges the authors faced.

**We understand the reviewer’s concerns, as the quantity of data is in some ways overwhelming, we wanted to ensure that the approaches we took would use R packages and statistical approaches with plentiful online documentation, analogous to the approach we took in selecting hardware. Below we provide line-by-line responses describing changes we made in line with their guidance to improve the MS.**

Introduction
Line 43: Provide subfamily of giant clams.

**We have added the subfamily.**

Line 58: If they are above their maximum thermal tolerance, wouldn’t they be dead and therefore open? Do you mean when they are approaching their maximum thermal tolerance?

**Yes, you are correct. We have revised that sentence to note they are approaching their tolerance.**

Line 59: Doesn’t “The clams close…to minimize exposure to ultraviolet radiation…” contradict the previous statement that they bask wide-open during the daylight hours to optimize photsynthetic production? Daytime is when they would be exposed to UV radiation.

**The paper cited for this sentence used UV-emitting bulbs over cultured clams in aquaria to simulate the conditions intertidal clams would experience. Giant clams generally bask in the daylight hours, but the wild clams that were studied were subtidal and likely did not experience excessive UV light. This is likely only a behavior observed intertidal populations. The giant clams in Biosphere 2 did not experience tidal emersion and were protected from almost all UV light by the overhead glass dome of the structure. We have added these details to the text for clarification.**

Lines 72-73. Use of electrodes calibrated by a specific formula and connected to a waterproof wireless transmission box would not preclude use of valvometric monitoring in aquaculture. What is the actual problem? The expense of these systems? If so, then say this plainly.

**The use of a calibrated formula would require additional preparation before data collection, adding to the time and expense of further studies. The wireless transmission equipment would also add to the expense and reduce reliability of the system. We have elaborated on these concerns in the text.**

Lines 74-75. “…close-source resources..” Same question why is this more of a problem for the developing world? Expense? Proprietary issues?

**Yes, the key concern would be expense and reproducibility. Especially during the pandemic, supply constraints have reduced availability of many types of electronic components, which has been a challenge in other aspects of our research. The advantage of the Raspberry Pi and Arduino systems is that there are many nearly-equivalent alternatives and “clones” available for each type of hardware. Since the publication of this manuscript, Raspberry Pi devices have become backordered, but the availability of alternatives would allow researchers to replicate the results using different brands. With a proprietary system, that is not an option. We have added mention of these advantages in the text.**

Lines 80-84. Why is the use of hot glue or air-curing adhesives considered “invasive”. These are external attachments, not internal (i.e. invasive) attachments. Is the real problem the length of exposure to air during drying rather than ‘invasivness’? Do hot glue or “other air-curing adhesives” require a longer curing time than the Coral Frag Glue used in this study to attach small magnets? It seems to me that this study used very similar, external attachment techniques as previous studies. Did the previous study techniques require an exposure time that was longer than 10 minutes? How much longer?

**The reviewer is right that the better, more specific term would be “disruptive” rather than invasive. Hot glue generally requires 20 minutes to set and 24 hours to fully cure. Coral frag glue can set underwater after 5 minutes in air. Another issue was the lack of documentation of what attachment method was used for prior studies of giant clams. We felt it was important to provide excessive detail in our method. We’ve added mention of approaches other papers took where available.**

You say these are intertidal animals and can survive hours of exposure, often with shells open. Is reducing air exposure to < 10 minutes really needed for an animal that has evolved to live in an environment with regular, periodic exposure cycles?

**While giant clams can live intertidally, we felt it was important to replicate natural conditions wherever possible. Sudden complete emersion was only experienced 1-2 previous times by the clams used by this study, which were cultured in 1-2 feet of water. We wanted to ensure that we did not take experimental steps which would lead to stress responses that affected the data and led to prolonged closure. We also wanted to reassure concerned readers that we considered the clams’ welfare in our methods. Some aquarists are under the mistaken impression that air exposure is fatal to giant clams, and we wanted to address any such misconceptions head on while not belaboring the point. We have added mention of these points to the text.**

Line 91-93. Did both “attachments” cure underwater? I understand the epoxy cured underwater, but did the Coral Frag Glue also cure underwater? How did use of Coral Frag Glue and underwater epoxy allow for easy detachment? Are they weak glues? Weaker than the hot glue and other air-curing adhesives previously referred to? I don’t necessarily doubt the authors when they say their attachments represent an improvement, but strongly suggest more detail to support this claim. I have dealt with very similar problems/issues with adhesives and some information regarding/quantifying the actual improvements in air drying times and ease of removing glued sensors compared to previous adhesives would be very useful.

**Yes, the coral glue also cures underwater. It is an altered cyanoacrylate formulation with a jelly-like consistency. We found the combination of epoxy and glue to be ideal for attaching the silicone-coated Hall sensor to the shell. But the epoxy was also removable with around 2-3 lbs of force applied by a pry action from a Philips head screwdriver. We had quite a bit of challenges finding the right attachment method during the experiment. We found epoxy was good at attaching to the shell, but not the probe, while the frag glue was great at attaching to the probe, but not the shell. When we combined the two, we had a breakthrough. We have added details of these points to the text.**

Methods
Overall, really nice description of sensor methodology and troubleshooting! Much of this section is very clear with a lot of useful information. Just a few concerns
Lines 116-118. So…were the supplementary LED lights strong enough to bring the solar PAR above 200 umol? It is a good idea but did it work? Supplemental Fig 2 (cited in text) suggests it did work for a few hours but the legend suggests this pattern is based on an assumption of a peak value of 370 umoles rather than what was actually achieved. However Fig. 2b provides direct measurements that suggest the LED lights were successful in achieving the goal of >200 umol. Authors should clearly state whether the goal was attained and (I think) cite Fig 2b as evidence.

**Yes, we achieved far in excess of 200 umol, particularly in the morning hours when the sunlight and LED light was combined. We added a mention of the figure discussing direct PAR levels that the clams experienced and thank the reviewer for the suggestion. We felt it was important to include information on the PAR levels coming from the lights to compare to levels mentioned in prior studies of captive giant clams, which were on the dim side compared to what is recommended by *Tridacna* culture professionals.**

Lines 191-195. Why use two different glues? Does the Coral Frag Glue produce volatiles? If so, why not just use the underwater epoxy for attachment of the magnets.

**As stated above, we had trouble with attachment via one or the other type of adhesive for the probe side, but for the magnets, we found the frag glue worked fine. We have made that clearer in the text.**

Lines 213 – 222. Authors do a nice job of explaining how Z-scores and subsequent Percent closure was calculated, although they say “Percent closure was calculated with the formula:” and the following formula calculates percent open…not percent closure.

**We have changed all mentions in the text (and the equation) to read “percent closed”.**

Why are results in the figures reported in terms of Zscores and not percent closure? Percent closure is much easier for the reader to understand and interpret. I would strongly suggest reporting % closure rather than Z-scores for most of the figures.

**We used z-scores in most of the text because it added only one level of processing to the data in the calculation of the variable. We were concerned that conversion to percent open would add noise regarding the assumptions involved in the variables of the equation. However, in the interest of easy interpretation, we have remade key figures with percent closure rather than Z-score values.**

Lines 222-23. Authors need to better explain how “closure events” were defined and calculated. These are very important issues in valveometry and the definition and calculations often differ among studies. Were the authors defining a closure event as a period when % open remained below a specific threshold (i.e. <50%? <10%?), or do they define a closure event as a period of sequential declines in “% open” regardless of what the actual % is (i.e. a decline from 99% to 90% open would be considered a closure event), or a trough within some sort of wave function fit to the % open data? Some other definition? It is not clear how a “..local maxima/minima in a 3-point (>15 second) moving window.” is used to define “closure” and what “closure” actually means. I don’t doubt that they are using a valid technique, they just need to better explain what a closure event is in the context of this study and how it is calculated. This is important because many readers not familiar with valveometry will take “closure event” literally and assume that this means the shell is completely “closed”, especially when the authors say “..in terms of …before reopening.” I do not think a closure event refers to full closing and reopening of the valves in this study.

**The reviewer is right that this is an important distinction. We were more interested in measuring the valve movement in all its forms, which involved identifying points where the valves became less open and stayed less open for two successive measurements. This aligns with the typical behavior of the clams, which retract their mantles and do not typically immediately “unfurl” after a closure. We settled on the three-point window based on our visual observations of this pattern. We have added additional information on these specifics and the rationale to the text.**

Results:
Lines 246-261. It is very difficult see the patterns the authors are describing from the figures. Here are some examples.
Line 246: “All three giant clams in this study showed patterns of opening in the hours leading to sunrise, typically around 3-4 Am (Fig 2A).” Figure 2A shows data from only a single clam. No times are provided in Fig 2A, making it very difficult to assess whether this is true.

**Supplemental Figure S3 does show the diurnal pattern across all three clams. We have inserted a reference to that figure in the main text. This diurnal opening was the general pattern observed across all individuals. We have made the axis marks every 6 hours in Fig. 2 and 3 to allow times to be better discerned.**

Line 251 “..reduced incidence of closure during peak daylight hours (4 AM -2 pm), when light was above…”. This time frame does not seem to represent PEAK daylight hours. LED lights did not turn on until 8 A.M. No information is given regarding sunrise, but from what I can tell, the sun doesn’t even rise until ~5:30-7:30 in Arizona, depending on the time of year.

**As is visible in Fig 2, the light reaches a peak in the late morning, when the sun is at an angle allowing direct insolation of the clams. By the early afternoon, the sun has retreated behind trees overhanging the Biosphere 2 ocean. The clams seemed to follow this trend in their behavior. We have added specificity to the text regarding this dynamic.**

Line 254 “..researcher presence..close when..” here and throughout, it is unclear whether daylight closures in response to shadows from researcher presence is simply speculation or something that was actually measured.

**This was directly observed by the researchers. The clams close when shadowed from overhead to protect themselves as they have simple eyes. This behavior has noted in the prior literature cited in the introduction. We added additional citations to such studies. We observed that most of the late-morning closures in the records aligned directly with our “visits” to the clams. While we did not take detailed logs of each visit, we felt it was important to note to explain such closures, which might not occur in clams not shadowed above by snorkeling researchers.**

Lines 257-261. Closure percentage means are provided in text but % closure data is not actually shown in the Fig 3 (Z-scores), Fig 4A (# closures), or Supplemental Fig 3 (Z-score). Variance around the means (which was apparently high) is not provided, and there was no statistical analysis testing for differences in % closure between high and low light periods making it very difficult to assess whether mean low-light closures (30%) were really different from mean higher-light closure (12.7%).

**We have added some additional descriptive statistics and pairwise tests quantifying the difference between lighted and unlighted periods to the text (after around line 286).**

Lines 276-285. This section describes “valve clapping” results – frequent rapid valve closure events and compares between lighter and darker time periods. Only one figure (Fig 4A) is referenced here and it describes # closures/day. How closure frequency differs between time periods within a day (i.e. light/dark periods) cannot be discerned from this graph. There is no supporting analysis or data for the statement “..closed once every 34 minutes during darker hours, but only once every 76 minute during lighter hours.” There is weak support for the duration of closures differing between lighter and darker hours (63 vs 90 seconds) in Fig. 2A but this figure is not referenced here and even if it was, it would be difficult to see this pattern. More importantly, as in the previous comment, there is no actual analysis testing whether duration differed between lighter and darker hours, and no indication of the variation around the means of closure duration (63 vs 90 seconds). With regards to the final statement “…no significant long term trends in closures per day were observed…with marked variance around the mean of 34 closures per day (Fig. 4A): Was this data actually analyzed to see if there was a long term trend or did the authors just look at the data and conclude there was not trend. The variance around the mean of 34 closures per day could easily be calculated. What was it?

**The reviewer is right that additional statistical tests are merited to quantify and verify the descriptive statistics we used in those lines. As mentioned above, we have expanded this section to be more formal, quantitative and less anecdotal.**

Lines 286-291. This section is much better. Authors analyzed data using a GAM approach and showed a significant relationship between daily closure frequency and pH as well as log ChlA. They also used a CCF approach to show that valve closure frequency had a lagged response to ChlA but no to pH. My only concern is that it is difficult to interpret Fig 5 because it is not clear what the Y-axis represents. The legend simply says “The s(x) terminology of the y axes refers to the output being smoothed.” It is not clear to me what the Y-axis represents and I suspect it will not be clear to most readers.

**The “s(x)” function terminology is standard for the both of the major GAM R packages. We added elaboration on this in the caption to note that the smoothing in this case refers to the application of a series of splines, or component functions applied over limited spaces. Each spline can be weighted and applied additively to fit the response variable (closure rate) to each of the explanatory variables, accounting for nonparametric effects and differing distributions. Each spline-based curve explains a certain proportion of the variance of the response variable, with some like pH and chlorophyll-a demonstrating statistical significance and others like light not reaching significance. We have also added a reference to a foundational paper on the topic of GAMs from Hastie and Tibshirani.**

Lines 291 – 300. I have to admit, I am totally lost in this section. I had a lot of difficulty interpreting this text and the corresponding graphs.

**Wavelet decomposition is not a commonly used approach in the previous valvometric literature, but we felt it had much to contribute in revealing periodic elements of the data, which seemed of interest to Reviewer 2. We have added mention to the discussion of why we felt this unconventional visualization method was useful and included a general reference on how wavelets work.**

Discussion.
Given the concerns listed for the Results section, I did not spend a lot of time reviewing the Discussion because it was not always clear which results/conclusions were strongly supported by the data and analyses and which were just speculative. My major suggestion would be to cut the length of the discussion, focusing on pros and cons of this low-cost approach and on the results that are clearly supported by the data. Authors should greatly reduce the speculative discussion and instances of circular logic.

**We have simplified our discussion where appropriate. Thank you for the helpful suggestions here and above.**

Reviewer #2: This manuscript is well written and deals with the development of low cost equipment to measure the behavior of bivalves by valve spacing. The valve closures and openings show a very clear daily cycle. The statistical treatments here are convincing (cycles at 6, 12 and 24 h, wawelet analysis) It is really interesting. The duration of the experiment is long and this is also an excellent point.

**We are encouraged that the reviewer appreciates the wavelet approach in analysis of the data, as well as the general design of the experiment.**

However the results are much clearer on a daily scale than on a monthly scale. I can't understand why

**Generally, we expected stronger trends during the summer than we saw. As mentioned in the discussion around line 435, we suspect that this may be due to the general lack of strong seasonal changes in light or temperature during the study period, which may have differed if the experiment was conducted during the declining light of autumn, or increasing light of spring months. We have added mention to the discussion of those potential opportunities for further research.**

I don't understand how valvometry informs on the resilience of a coral mass (?).

**We believe that the coral “mass” the reviewer is referring to is the health of the reef as a whole? If so, as with prior valvometric studies in other regions, we believe that this behavioral data can provide important live-collected information on how animals are responding to environmental dynamics on short-term timescales, in an individual and group context. Such data is not easy to collect for photosymbiotic corals, which are difficult to study in terms of behavior. We believe that the data in this study shows how giant clam behavior relates to external productivity, light levels and pH, all of which are factors influencing photosymbiotic organisms, and therefore coral reef health worldwide. We’ve added elaboration on this point to the conclusion.**

Am I supposed to know Biosphere 2?

**We included a description of the Biosphere 2 ocean in the introduction to provide context for readers unfamiliar with the layout and history of the facility. If the reviewer feels more (or less?) information is needed, please provide more information on what changes we can make.**

Despite the explanations of the authors, I do not understand why the proposed method is non-intrusive since it is a matter of taking a clam out of its environment. I do not understand how it will be made autonomous.

**Reviewer 1 had similar questions, and so we have made our description more specific, describing the methods involved as “less disruptive.” There is indeed the unavoidable stressor involved in removing a clam from its natural environment, but we feel the data still shows natural-type behavior in the studied clams, compared to the Schwartzmann et al.’s previous valvometric study of wild giant clams. We are unclear of what is meant by “autonomous”, but we have added greater mention to the text of how we assert this system is low maintenance, once configured.**

The hall effect sensors can be calibrated since the interval distance is measurable and a range can be achieved at the end of the experiment. The experimentation is done in a giant tank but it seems that the electronics stayed out of the water.

**Yes, the electronics stayed out of the water, in a waterproof gasketed junction box, as described around line 168, though that is an optional accessory and other methods of covering would likely suffice, as the Raspberry Pi and Arduino proved resilient to the high humidity of the Biosphere 2 environment, as long as they were protected from salt water. We have added mention of the housing to the text.**

I did not understand the light variations presented in figure 2. The PAR measurement (why a planar sensor?) increases abruptly when you turn on the light.

**The rapid increase in the plotted record in the late morning was not just due to the LED lights turning on, but also the simultaneous direct insolation by the rising sun, which only rose high enough to be above the retaining wall next to the clams after around 7-8 AM (see the new addition to supplementary figure 2). We used a planar sensor because it was more applicable to understanding the insolation the clams experience on the surface of their flat mantle surfaces. A spherical sensor would measure PAR from all directions, including below, which would not be relevant to insolation of the clams. We have added description of this to the text.**

What about the PAR decrease during the rest of the day? Why does the light suddenly increase at midnight? Note that this increase seems to induce a change in the behavior of the clams.

**As mentioned in the text, the light declines in the early afternoon as the sun falls behind trees overhanging the Biosphere 2 ocean. The reviewer is astute to note the spike in measured light at midnight some nights. We do not have a complete explanation of what that data point is, but we can confirm from direct observation that there is no flash of light from the lighting rig at midnight. Our best explanation is a software bug in the LI-1500 light logger, which is not designed for continuous underwater collection. We have added description of this to the figure caption.**

You state that the pH of the sea water is influenced by primary production alone. No. Calcification, respiration, and many other processes influence this parameter.

**We did not seek to imply that pH is only a function of production, and have made that clearer with a mention around line 425. Respiration is indeed a major factor influencing pH of the Biosphere 2 ocean, while calcification is currently less so, due to the current small population of calcifying organisms in the tank.**

The legend to your figures is in the body of the text. I do not agree with this inovation.

**That was the instruction provided by the PLOS journal, and will be corrected upon publication.**

Do you have proof of the presence of predatory worms Harmodice by video, counting, and direct observation ?

**We have included a picture of a *Hermodice* worm in the supplement. We don’t have video, but prior surveys have mentioned their presence in the Biosphere 2 ocean, so we added a reference to one study, and they are a well-known pest in the reef aquarium industry.**

Closure and opening rhythms could be linked to oxygen concentration in seawater in particlar within the paleal cavity
**This would be an interesting subject for further research! While we collected fluid from the extrapallial space (EPF), we did not measure in-situ oxygen concentration of that fluid. Prior studies have shown how giant clams can increase the O_2_ concentration of aquarium tanks during sunlit hours, and such dynamics would be interesting to explore in future work.**

Translated with www.DeepL.com/Translator (free version)

6. PLOS authors have the option to publish the peer review history of their article ([what does this mean?](https://journals.plos.org/plosone/s/editorial-and-peer-review-process#loc-peer-review-history)). If published, this will include your full peer review and any attached files.


If you choose “no”, your identity will remain anonymous but your review may still be made public.


**Do you want your identity to be public for this peer review?** For information about this choice, including consent withdrawal, please see our [Privacy Policy](https://www.plos.org/privacy-policy).

Reviewer #1: No

Reviewer #2: No

[NOTE: If reviewer comments were submitted as an attachment file, they will be attached to this email and accessible via the submission site. Please log into your account, locate the manuscript record, and check for the action link "View Attachments". If this link does not appear, there are no attachment files.]

While revising your submission, please upload your figure files to the Preflight Analysis and Conversion Engine (PACE) digital diagnostic tool, <https://pacev2.apexcovantage.com/>. PACE helps ensure that figures meet PLOS requirements. To use PACE, you must first register as a user. Registration is free. Then, login and navigate to the UPLOAD tab, where you will find detailed instructions on how to use the tool. If you encounter any issues or have any questions when using PACE, please email PLOS at [figures@plos.org](mailto:figures@plos.org). Please note that Supporting Information files do not need this step.

*In compliance with data protection regulations, you may request that we remove your personal registration details at any time.* [*(Remove my information/details)*](https://www.editorialmanager.com/pone/login.asp?a=r)*. Please contact the publication office if you have any questions.*
